# Compound 49b Restores Retinal Thickness and Reduces Degenerate Capillaries in the Rat Retina following Ischemia/Reperfusion

**DOI:** 10.1371/journal.pone.0159532

**Published:** 2016-07-20

**Authors:** Li Liu, Youde Jiang, Jena J. Steinle

**Affiliations:** 1 Department of Anatomy and Cell Biology, Wayne State University, Detroit, MI, 48201, United States of America; 2 Department of Ophthalmology, Wayne State University, Detroit, MI, 48201, United States of America; Indiana University School of Medicine, UNITED STATES

## Abstract

We have recently reported that Compound 49b, a novel β-adrenergic receptor agonist, can significantly reduce VEGF levels in retinal endothelial cells (REC) grown in diabetic-like conditions. In this study, we investigated whether Compound 49b could protect the retina under hypoxic conditions using the ischemia-reperfusion (I/R)-induced model in rats, as well REC cultured in hypoxic conditions. Some rats received 1mM topical Compound 49b for the 2 (5 rats each group) or 10 (4 rats in each group) days post-I/R. Analyses for retinal thickness and cell loss in the ganglion cell layer was done at 2 days post-I/R, while numbers of degenerate capillaries and pericyte ghosts were measured at 10 days post-I/R. Additionally, REC were cultured in normal oxygen or hypoxia (5% O_2_) only or treated with 50 nM Compound 49b for 12 hours. Twelve hours after Compound 49b exposure, cells were collected and analyzed for protein levels of insulin-like growth factor binding protein 3 (IGFBP-3), vascular endothelial cell growth factor (VEGF) and its receptor (KDR), angiopoietin 1 and its receptor Tie2 for Western blotting. Data indicate that exposure to I/R significantly decreased retinal thickness, with increasing numbers of degenerate capillaries and pericyte ghosts. Compound 49b treatment inhibited these retinal changes. In REC cultured in hypoxia, levels of IGFBP-3 were reduced, which were significantly increased by Compound 49b. Hypoxia significantly increased protein levels of VEGF, KDR, Angiopoiein 1, and Tie2, which were reduced following Compound 49b treatment. These data strongly suggested that Compound 49b protected the retina against I/R-induced injury. This provides additional support for a role of β-adrenergic receptor actions in the retina.

**Highlights**

—Compound 49b prevented I/R-induced retina damage
—Compound 49b significantly reduced VEGF levels in retinal endothelial cells (REC) grown in hypoxia
—Compound 49b significantly reduced Angiopoietin 1 levels in REC grown in hypoxia

## Introduction

The numbers of people worldwide with diabetes is reaching epidemic levels. Within 20 years of diagnosis of diabetes, approximately 30% of patients will have some symptoms of diabetic retinopathy (ADA website). While anti-VEGF therapies are effective in some patients with diabetic macular edema and proliferative diabetic retinopathy, additional treatment modalities are needed. We have reported that our novel β-adrenergic receptor agonist, Compound 49b, can significantly protect the retina against diabetes-induced functional, neuronal, and vascular damage [1]. This protection is mediated, at least in part, through increasing IGFBP-3 levels in the diabetic retina [2]. We also recently demonstrated that Compound 49b significantly reduced VEGF levels in REC cultured in high glucose through an IGFBP-3-mediated pathway [3].

In order to investigate whether Compound 49b is effective in additional models of retinal damage, we employed the ischemia/reperfusion (I/R) model of acute ischemia-induced retinal damage. This model has been used to demonstrate that minocycline can reduce retinal inflammation and permeability [4, 5]. Similarly, work using healthy and diabetic CD34+ stem cells demonstrated that only healthy cells can repair damaged vessels in the I/R-exposed retina [6]. To support the work in the I/R rat retina, we investigated the effects of hypoxia on retinal endothelial cells (REC), focusing on the role of Compound 49b in the regulation of VEGF and angiopoietin 1 signaling. We chose to focus on VEGF and angiopoetin 1, as we had previously reported that loss of sympathetic neurotransmission significantly increased VEGF, KDR (VEGF receptor 2), angiopoetin 1 (Ang1) and angiopoetin 1 receptor (Tie2) mRNA in the rat [7]. Others have also investigated the role of hypoxia on angiopoetin 1 in the OIR model [8]. Additionally, work has been done on retinal pericytes to show that hypoxia and VEGF can both activate angiopoetin 1 [9]. Thus, we wanted to investigate whether Compound 49b could regulate VEGF and angiopoetin levels in REC grown in normoxia vs. hypoxia.

While we were investigating hypoxia-induced changes in VEGF and angiopoetin 1, we also were interested in the role of hypoxia on insulin-like growth factor 1 binding protein 3 (IGFBP-3) in REC. IGFBP-3 is a hypoxia-inducible gene [10]; however much of this work has been done in cancer models. There is additional information that hypoxia can drive IGFBP-3 synthesis in cardiovascular cells and myocardial infarction models [11][12]. However, we have found that diabetes decreases IGFBP-3 levels, which was associated with increased TNFα levels [1]. Others have also reported that IGFBP-3 can increase pericyte ensheathment, as well as reduce microglial activation in the hypoxic phase of the OIR model [13]. Thus, the responses of IGFBP-3 to hypoxia may be tissue- or disease specific.

In this study, we wanted to investigate the role of Compound 49b in an acute ischemia model, using the I/R model. Additionally, we studied the role of Compound 49b on protein levels of known angiogenic proteins exposed to a hypoxic insult.

## Materials and Methods

### Rats

All animal procedures meet the Association for Research in Vision and Ophthalmology requirements and were approved by the Institutional Animal Care and Use Committee of Wayne State University (A-11-09-14) and conform to NIH guidelines. Forty male Lewis rats were purchased from Charles River at 8 weeks of age. Rats were allowed to acclimate to the vivarium at Wayne State University for 1 week prior to initiation of treatments. Once experiments were completed, animals were euthanized using an overdose of ketamine and xylazine, followed by opening of the chest cavity to stop the heart.

### Ischemia/Reperfusion Model

Animals were anesthetized with an intraperitoneal injection of ketamine and xylazine. Once animals did not respond to toe pinch, the anterior chamber of the eye was cannulated with a 32-gauge needle attached to an infusion line of sterile saline. The eye was subjected to 90 minutes of hydrostatic pressure (80-90mmHg, TonoPen, Medtronic, Jacksonville, FL) to induce retinal ischemia as evidenced by blanching of the iris and loss of red reflex [6]. After 90 minutes, the needle was withdrawn and intraocular pressure normalized, causing reperfusion. The contralateral eye served as an in-animal control. Once experiments were completed, animals were overdosed by ketamine and xylazine, followed by opening of the chest cavity to stop the heart.

### Treatments

In some groups, Compound 49b was given as an eye drop (1mM, 4 drops/eye) as soon as the I/R procedure culminated and continued daily (up to 10 days) until animal sacrifice.

### Retinal Thickness

Two days after I/R exposure, 5 rats in each group of rats were sacrificed for measurements of neuronal thickness, as we have done previously [1]. Briefly, the whole eye is removed and placed into formalin. The retina is separated from the rest of the globe and embedded in O.C.T. freezing medium. Ten micrometer sections were then made from throughout the retina, followed by toluidine blue staining. Analyses were done on 10 sections from 5 animals in each group as we have done in the past [1, 14].

### Vascular analyses

Ten days after I/R exposure, the remaining rats (5 from each group) were sacrificed to measure degenerate capillaries and pericyte dropout. Briefly, the experimental and control globes are placed into 4% paraformaldehyde for 10 days [15]. Retina were dissected out and rinsed in water overnight. The retina was then transferred into an elastase solution (40ul/ml) and incubated at 37°C for 2.5h, followed by activation of the elastase in Tris-HCl buffer (pH 8.5) for an additional 12 hours [15]. The glia and the neuronal regions were gently brushed away. Once only the retinal vasculature was present on the slide, the flatmount was stained with periodic-acid shiff to allow for measurements of degenerate capillaries and pericyte ghosts [1, 15, 16]. Regions from four retinal flatmounts were counted in each group of animals for pericyte ghosts and degenerate capillaries at ~300μm from optic nerve. Examination was done on 1000 capillary cells (endothelial cells and pericyte) in those 4 fields to count pericyte “ghosts”, excluding any pericyte ghosts observed on degenerate capillaries. Counts were done at 40x using a Leica Brightfield Microscope.

### Retinal endothelial cells

Primary human retinal endothelial cells (REC) were obtained from Cell Systems Corporation (CSC, Kirkland, Washington). Cells were grown in Cell Systems medium supplemented with microvascular growth factors, 10ug/mL gentamycin, and 0.25ug/mL amphotericin B (Invitrogen, Carlsbad, CA). Once cells reached confluence, some dishes were placed into a hypoxic chamber with O_2_ levels set at 5% (Hypoxia). Cells were allowed to equilibrate at the lowered oxygen levels for 24 hours and some dishes were then treated with Compound 49b at 50nM for 12 hours. Additional cells were maintained at normal oxygen levels (Normoxia) only or treated with Compound 49b at 50nM for 12 hours.

### Western blotting

After appropriate treatments and rinsing with cold phosphate-buffered saline, cell lysates were collected in lysis buffer containing protease and phosphatase inhibitors and scraped into tubes. Protein extracts were prepared by sonication. Equal amounts of protein from the cells were separated on pre-cast tris-glycine gel (Invitrogen, Carlsbad, CA), blotted onto a nitrocellulose membrane. After blocking in TBST (10mM Tris-HCl buffer, pH 8.0, 150 mM NaCl, 0.1% Tween 20) and 5% (w/v) BSA, the membrane was treated with appropriate primary antibodies followed by incubation with secondary antibodies labeled with horseradish peroxidase. Antigen-antibody complexes were detected by chemilluminescence reagent kit (Thermo Scientific). Primary antibodies used were KDR (Cat# 2472, Cell Signaling), Angiopoetin 1 (Cat# MAB293, R&D Systems), VEGF (Cat# BAF293, R&D Systems), Tie 2 (Cat# 42–5100, Zymed), and IGFBP-3 (Cat# 09–180 EMD Millipore). All antibodies were used at a dilution range from 1:200 to 1:1000, depending on each specific antibody and company recommendations. Chemilluminescence was measured on a C500 Azure Series system (Azure Biosystems, Dublin, CA). Densitometric analyses were done as a ratio of the protein of interest to β-actin (Cat# MA5-15739-HRP, Invitrogen).

### Statistics

Statistical analyses were done using 1-way ANOVA analyses with Student Newman Keul’s post-hoc testing in Prism software (GraphPad, San Diego, CA) for the animal studies. Non-parametric Kruskal-Wallis with Dunn’s post-hoc tests were used for the cell culture data. P<0.05 was considered statistically significant.

## Results

### Compound 49b restores retinal thickness in rats exposed to I/R

**Fig 1:**
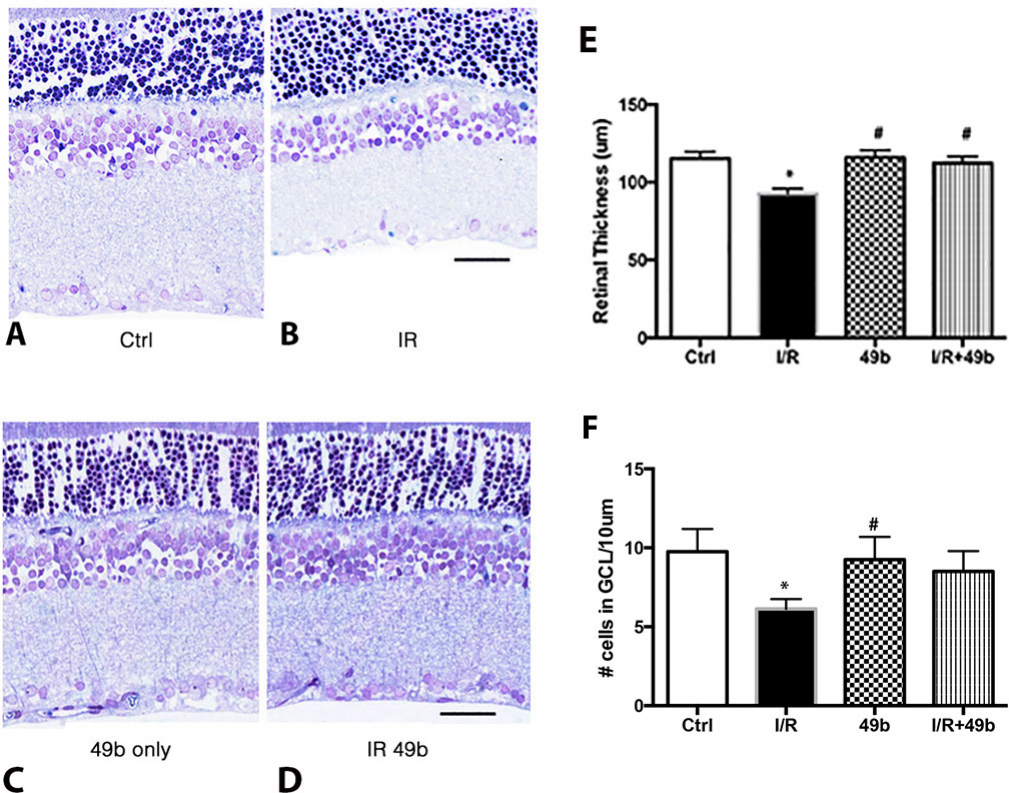
Compound 49b can overcome ischemia/reperfusion-induced decreases in retinal thickness. Panel A represents the control (left eye). Panel C represents eyes treated with 1mM Compound 49b only. Panels B, D are eyes exposed to ischemia/reperfusion (B) or I/R+Compound 49b (D). Panel E provides a graphical demonstration of the mean±SEM for retinal thickness in each group. Panel F provides a graph for the numbers of cells in the ganglion cell layer. Data is mean±SEM. N = 5 for each group. *P<0.05 vs. control; # P<0.05 vs. I/R only without Compound 49b.

We have previously reported that Compound 49b could reduce the loss of retinal thickness and cell number in the ganglion cell layer (GCL) in the diabetic rat retina [1]. Following 2 days of exposure to I/R, the retina is significantly thinner with fewer cells in the GCL compared to the eye left unexposed (Fig 1B vs. 1A). No change in retinal thickness was noted in eyes treated with Compound 49b+I/R versus eyes treated with Compound 49b only (Fig 1D vs 1C). While there were more cells in the GCL in the I/R+Compound 49b group compared to I/R only, it did not reach statistical significance. Panel E provides graphical data of mean ± SEM for retinal thickness, while panel F provides the data for cell numbers in the GCL of eyes. This suggests that Compound 49b can prevent loss of retinal thickness and improve numbers of cells in the GCL in the retina following exposure to I/R.

### I/R results in vascular damage, which is reduced with Compound 49b treatment

**Fig 2:**
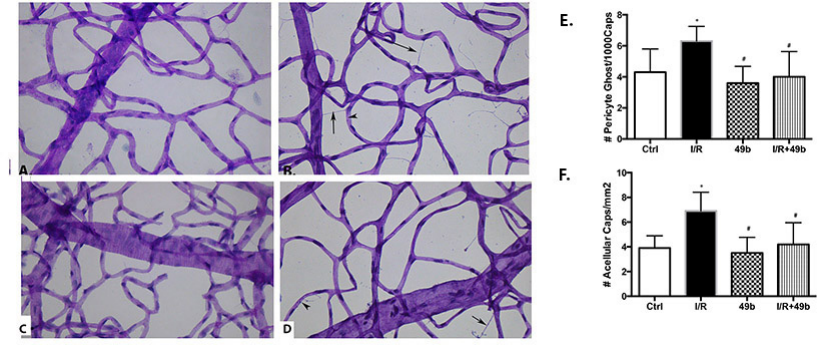
I/R produced significant vascular damage that were reduced following Compound 49b treatment. Panel A represents the control (left eye). Panel C represents eyes treated with 1mM Compound 49b only. Panels B, D are eyes exposed to ischemia/reperfusion (B) or I/R+Compound 49b (D). Panel E provides a graphical demonstration of the mean±SEM for pericyte ghosts in each group. Panel F provides a graph for the numbers degenerate capillaries. Arrows represent degenerate capillaries and arrowheads are pericyte ghosts. Data is mean±SEM. N = 4 for each group. *P<0.05 vs. control; # P<0.05 vs. I/R only without Compound 49b.

Similar to the data on retinal thickness and cell numbers, we also evaluated degenerate capillary and pericyte ghost numbers in the retina following exposure to I/R and Compound 49b+ I/R. Fig 2B vs 2A demonstrates that exposure to I/R significantly increased numbers of degenerate capillaries and pericyte ghosts when compared to the unexposed contralateral eye. Pericyte ghosts and degenerate capillaries were reduced by Compound 49b treatment after I/R exposure (Fig 2D vs 2C), as we reported for diabetic rats [1]. Quantitation of means of the degenerate capillaries is provided in Fig 2F and 2E provides counts of pericyte ghosts.

### Compound 49b significantly increases IGFBP-3 in REC grown in hypoxia

**Fig 3:**
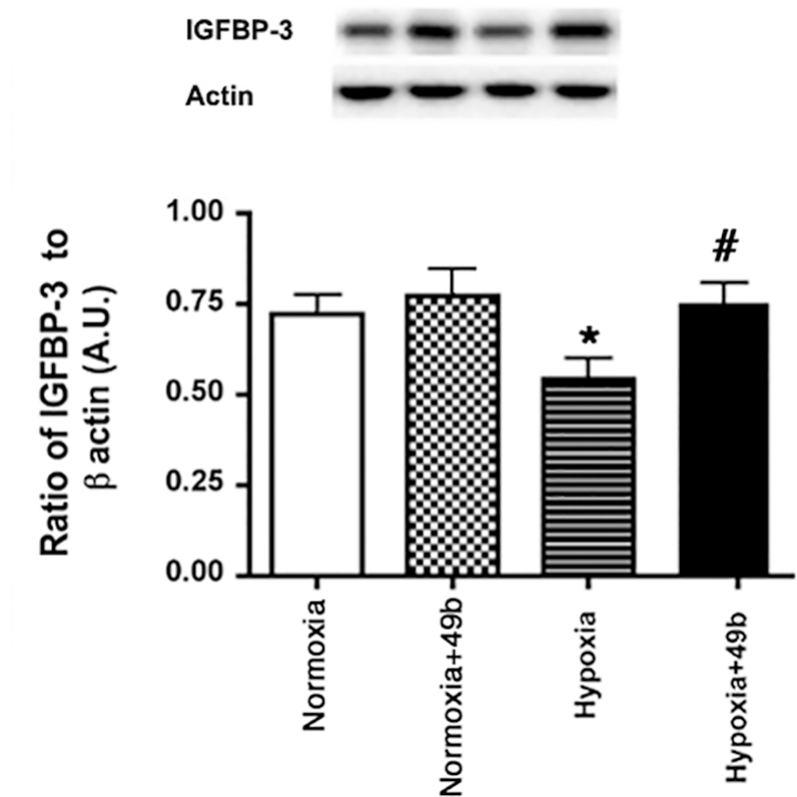
Protein levels of IGFBP-3 from retinal endothelial cells cultured in normoxia or hypoxia only and cells in each condition treated with Compound 49b. Hypoxia reduced IGFBP-3 levels, which were restored after treatment with Compound 49b. Data is mean ± SEM. *P<0.05 vs. normoxia. ^#^P<0.05 vs. hypoxia. N = 4.

We have shown that Compound 49b significantly increases IGFBP-3 in the diabetic retina and in REC cultured in high glucose [1]. We wanted to ascertain whether Compound 49b could regulate IGFBP-3 in REC cultured in normoxia or hypoxia. Data show that hypoxia significantly decreased IGFBP-3, which was reversed by treatment with Compound 49b (Fig 3).

### VEGF, angiopoietin-1, and their receptor levels are increased in hypoxia, which are reduced following Compound 49b treatment

**Fig 4:**
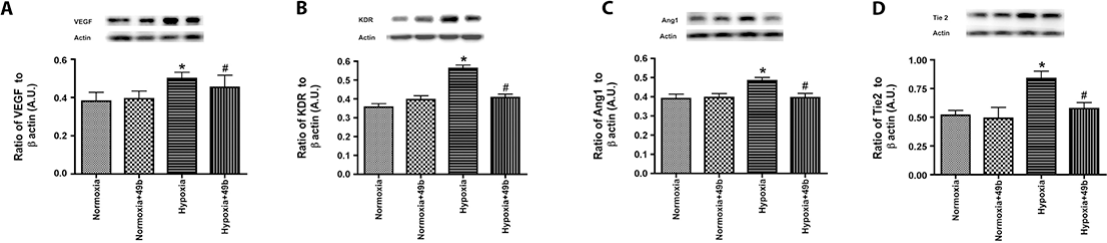
Protein levels of VEGF (A), KDR (VEGF receptor 2, B), Angiopoietin 1 (C) and Tie2 (D) from retinal endothelial cells cultured in normoxia or hypoxia only and cells in each condition treated with Compound 49b. Hypoxia increased protein levels of all proteins, which were significantly reduced after treatment with Compound 49b. Data is mean ± SEM. *P<0.05 vs. normoxia. ^#^P<0.05 vs. hypoxia. N = 4.

While there is literature to suggest that norepinephrine increases VEGF levels in tumor models [17, 18], others have reported that isoproterenol (a β-adrenergic receptor agonist) can reduce VEGF levels in the retina [19]. This suggests that β-adrenergic receptor agonist actions on VEGF appear to be tissue or cell type specific. Additionally, we recently reported that Compound 49b reduced VEGF levels in REC grown in high glucose [3]. We tested the effects of hypoxia on total protein levels of VEGF, Angiopoietin-1, KDR, and Tie-2. We found that hypoxia significantly increased all protein levels of all 4, as would be expected (Fig 4). Compound 49b significantly reduced protein levels of all 4 proteins to levels similar to baseline (Fig 4), suggesting that β-adrenergic receptor agonists may reduce angiogenic protein levels in hypoxic REC.

## Discussion

We have previously reported that Compound 49b, a novel β-adrenergic receptor agonist, could protect the diabetic retina against functional, neuronal, and vascular damage [1]. Additionally, we reported that Compound 49b could reduce VEGF levels through increased IGFBP-3 levels in REC grown in high glucose [3]. To expand those findings into an additional model of retinal disease, we employed the ischemia/reperfusion (I/R) model of retinal damage. Others have reported that this model replicated some of the neuronal damage associated with diabetes at 2 days post-reperfusion, while vascular damage was observed at 10 days post-reperfusion [4, 6]. Similar to our findings in the diabetic retina, Compound 49b was protective to the vasculature of the retina when administered after I/R. Compound 49b also restored retinal thickness to levels similar to retina not exposed to I/R; however, numbers of cells in the GCL were not statistically different in the I/R rat retina vs. those in the Compound 49b+I/R retina. A trend toward a Compound 49b-induced increase in GCL cell numbers is present but it does not reach statistical significance.

To further investigate the role of Compound 49b in models of ischemia, we treated REC with Compound 49b after exposure to hypoxia. The initial thoughts that β-adrenergic receptor agonists may regulate VEGF stemmed from findings that norepinephrine stimulates VEGF in a number of tumor models [17, 18]. Additionally, we have reported that loss of sympathetic neurotransmission significantly increased both VEGF and angiopoetin mRNA [7]. In REC, protein levels of VEGF and its receptor 2, KDR, were both increased following exposure to hypoxia, but reduced after Compound 49b. Work on bovine retinal pericytes has shown that hypoxia can significantly increase VEGF and angiopoetin 1 levels [9]. Our studies in REC cultured under hypoxic conditions showed that angiopoetin 1 and Tie-2 protein levels were increased, but returned to baseline levels by Compound 49b. Taken together, our data suggest that β-adrenergic receptor stimulation can reduce both VEGF and angiopoetin 1 levels. This response is likely cell type and disease specific.

In addition to significantly decreasing the pro-angiogenic factors VEGF and angiopoietin-1, Compound 49b also significantly increased levels of IGFBP-3. We have recently reported that Compound 49b can significantly increase IGFBP-3 levels, which are reduced after exposure to high glucose [1]. Others have also reported that IGFBP-3 can protect the ischemic retina through activation of endothelial cell precursor cells [19]. However, the reduction in IGFBP-3 in response to hypoxia in REC is contrary to work in H9c2 myocardial cells and esophageal squamous cell carcinoma xenografts where IGFBP-3 is increased in response to hypoxia [11][10]. However, these responses may be related to an activated cell type (cancer) vs. a relatively quiescent cell type (REC). Finally, IGFBP-3 can also regulate vasodilation and NO release, which could be protective to hypoxic REC [19]. Thus, additional studies are needed to further dissect out the role of IGFBP-3 in hypoxic REC.

Our findings of β-adrenergic receptor agonist’s protective effects on the I/R model are in contrast to findings for propranolol in the oxygen-induced retinopathy (OIR) model. Others have reported that β-adrenergic receptor antagonists are protective to the retina and reduce VEGF levels [20]. However, the same group did report that isoproterenol, a β-adrenergic receptor agonist, decreased VEGF levels in the oxygen-induced retinopathy model [21]; likely due to desensitization of beta-2-adrenergic receptors. In more recent work, the same group reported that topical propranolol, a β-adrenergic receptor antagonist reduced VEGF in the OIR retinal damage model [22]. Thus, the role of beta-adrenergic receptor agents on the retina may be disease specific and may involve a number of proteins. Furthermore, the work with topical propranolol was recently challenged by Chen et al (2012) as Chen et al. could not replicate the vascular protection in the OIR model after propranolol treatment [23]. The reasons for the differences between the Ristori findings and Chen’s findings are unclear, but they may be related to drug delivery, mice strain or other issues. Nonetheless, our findings of the effectiveness of Compound 49b, a β-adrenergic receptor agonist, administered topically to rats in the I/R model agrees with some literature and does not directly contrast any published work in other models of hypoxia.

## Conclusion

We used the I/R model to demonstrate that Compound 49b protected the retina against hypoxia-induced retinal damage. REC exposed to hypoxia had increased VEGF and angiopoetin 1 levels, which were reduced by Compound 49b. IGFBP-3 protein levels were reduced by hypoxic exposure to REC, which was restored to baseline by Compound 49b. These data provide an additional mechanism by which Compound 49b may protect the retina against neuronal and vascular damage.

## Abbreviations

I/R: ischemia/reperfusion
REC: retinal endothelial cell
VEGF: vascular endothelial cell growth factor
IGFBP-3: insulin-like growth factor binding protein 3
OIR: oxygen-induced retinopathy
GCL: ganglion cell layer
